# Consumption of Sugary Beverages by Adults Prior to Sugary Drink Tax in Colombia: An Analysis of the National Nutrition Survey 2015

**DOI:** 10.3390/nu18050716

**Published:** 2026-02-24

**Authors:** Michael Essman, Carlos R. Soto Díaz, Luis Carlos Forero Ballesteros, Mercedes Mora-Plazas, Luis Fernando Gómez, Lindsey Smith Taillie

**Affiliations:** 1 World Food Policy Center, Sanford School of Public Policy, Duke University, Durham, NC 27708, USA; michael.essman@duke.edu; 2 Department of Nutrition, Gillings School of Global Public Health, University of North Carolina at Chapel Hill, Chapel Hill, NC 27599, USA; csoto-diaz@unc.edu (C.R.S.D.); taillie@unc.edu (L.S.T.); 3 Instituto Nacional de Salud, Bogotá 111321, Colombia; 4 Departamento de Nutrición Humana, Universidad Nacional de Colombia, Bogotá 111321, Colombia; 5 Facultad de Medicina, Pontificia Universidad Javeriana, Bogotá 110231, Colombia; 6 Carolina Population Center, University of North Carolina at Chapel Hill, Chapel Hill, NC 27599, USA

**Keywords:** sugar-sweetened beverages, dietary intake, tax, Colombia, nutrition policy, Latin America

## Abstract

Background/Objectives: Colombia has implemented a tiered tax on sugar-sweetened beverages (SSBs), reaching 20% for high-sugar products by 2025. To inform evaluations of this policy, we estimated taxed and untaxed beverage intake prior to implementation and examined differences across sociodemographic groups. Methods: We analyzed 24 h dietary recall data from the 2015 National Survey of the Nutritional Situation of Colombia (ENSIN; *n* = 11,877 adults aged 18–64), which uses a stratified, multistage cluster sampling design covering all regions of the country. Beverages were categorized by 2025 tax thresholds: untaxed (<5 g added sugar/100 mL), taxed (≥5 g), and high-sugar taxed (≥9 g). Intake (mL, kcal) was estimated per capita and per consumer using survey-weighted two-part models, adjusting for sociodemographic characteristics. Results: Eighty-four percent of adults consumed taxed beverages, with per capita intake of 209 kcal (95% CI: 203–216) and 481 mL (95% CI: 464–497). Sixty-three percent consumed high-sugar taxed beverages, with intake of 134 kcal (95% CI: 130–139) and 292 mL (95% CI: 282–303) per capita. Untaxed beverages accounted for 109 kcal (95% CI: 106–112) and 837 mL (95% CI: 793–882) per capita. Across categories, the highest per capita kcal intakes were from untaxed dairy/milk substitutes (67 kcal, 95% CI: 64–69), any taxed fruit/vegetable juices (61 kcal, 95% CI: 55–66), and any taxed sodas/carbonated beverages (47 kcal, 95% CI: 44–50). Males consumed more taxed beverages of any type (255 kcal, 95% CI: 247–264) than females (174 kcal, 95% CI: 165–182). Conclusions: Prior to its SSB tax, Colombians had a high consumption of SSBs that would be subject to the tax. Future research should assess how consumption changed in response to the tax.

## 1. Introduction

Colombia faces a growing public health challenge, as the prevalence of adults who are overweight or obese increased from 45.9% to 56.5% between 2005 and 2015 [1]. In this same period, the prevalence of overweight and obesity among school-aged children in age groups 5 to 12 and 13 to 17 increased from 14.4% to 24.4% and from 12.5% to 17.9%, respectively [1]. Furthermore, disparities in obesity prevalence have emerged, with lower socioeconomic groups experiencing larger increases compared to their higher socioeconomic groups [2,3]. This rise in obesity is part of a broader nutrition transition occurring across Latin America, including Colombia, where there is a shift towards diets high in ultra-processed products (UPPs), including both foods and beverages [4].

Emerging research has found an inverse relationship between diets high in processed and ultra-processed foods and the nutrient quality of diets among Colombian schoolchildren from low- to middle-income families [5]. Within this context, a major concern is sugar-sweetened beverages (SSBs). A study utilizing data from the 2010 National Nutrition Survey in Colombia found that 79% of adults and 90% of children reported consuming SSBs [6]. These consumption patterns contribute to health inequalities, as exposure to SSBs is disproportionately higher among vulnerable populations [7]. Given the strong evidence linking SSB consumption to increased risks of obesity and type 2 diabetes, these patterns represent a significant public health challenge in Colombia [8,9,10].

In response, Colombia has introduced fiscal policies targeting the consumption of SSBs. In December 2022, Colombia passed taxes on SSBs and foods, which initially took effect in late 2023, with final implementation in January 2025 [11]. This measure imposes higher taxes on beverages with higher sugar concentrations. In the final phase of the tax, SSBs with 9 or more grams of added sugar per 100 mL are taxed at 20%, and those with at least 5 g but less than 9 g of added sugar per 100 mL are taxed at 12.2% [12,13]. Because Colombia’s tax is tiered by added sugar concentration, understanding baseline intake by tax-relevant thresholds and beverage types is essential for anticipating the tax’s population reach and for interpreting post-tax changes, including potential substitution and reformulation. Despite the growing concern about SSB consumption and the introduction of these fiscal policies, evidence on pre-tax beverage intake remains limited. Baseline data are needed on who consumes SSBs and which beverages contribute most to intake, including patterns by key sociodemographic groups and across beverage types aligned with the tax’s final sugar thresholds.

Previous studies have examined SSB consumption and disparities in Colombia, but they have not been designed around Colombia’s concentration-based tax thresholds or around estimating tax-relevant mean intakes. For example, prior analyses using food frequency questionnaire (FFQ) data reported the prevalence and frequency of sweetened beverage consumption [6]. More recent research also utilized FFQ data to examine consumption of beverage types and associations with adiposity, but was restricted to five urban cities, is not nationally representative, and was published prior to sugar concentration thresholds in the Colombian SSB tax policy [14]. The present study utilizes more recent data from 24 h recalls, which are generally more suitable than FFQs for estimating mean intakes in a population [15].

To our knowledge, no prior study has characterized pre-tax beverage intake in Colombia using the sugar-threshold categories defined in Colombia’s SSB tax law [12], alongside detailed beverage subcategories and population subgroups. This study aimed to address this gap by providing policy-relevant descriptive estimates of beverage intake among adults in Colombia prior to the implementation of the SSB tax. Using 24 h recall data from adults aged 18–64, we estimated (1) the prevalence of consumption, (2) mean intake volume (mL/day) and energy (kcal/day) reported both per capita and per consumer, and (3) the distribution of taxed and untaxed beverage intake overall and by beverage subcategory. For calculating taxed beverage intake, we used sugar concentration thresholds specific to Colombia’s SSB tax, and we examined intakes across the tax’s added-sugar concentration thresholds. We also assessed differences in these outcomes across key sociodemographic characteristics to support future tax evaluation and assessment of potential equity-related impacts.

## 2. Materials and Methods

### 2.1. Dataset

We used anonymized cross-sectional consumption data collected from the National Survey of the Nutritional Situation in Colombia (ENSIN 2015) [1]. Ethical approval for data collection was obtained by the original survey institutions, and no additional ethical approval was required for this secondary analysis (see Institutional Review Board Statement). This survey used a probabilistic multistage, stratified, cluster sampling design, aiming to have representative data from rural and urban areas, six regions, and 32 departments in Colombia [1]. Demographic information includes age, ethnicity, location, and socioeconomic status. Dietary data was collected by nutritionists via an in-depth 24 h dietary recall interview consisting of detailed questions about foods and beverages consumed the day before the interview. The total sample size of adults aged 18–64 was 11,877. Beverage data captured both industrialized sugar-sweetened beverages and beverages prepared at home.

### 2.2. Nutrient Profile Information and Categorization of Beverages

Added sugar content for each beverage reported in 24 h recalls was determined using three complementary approaches, corresponding to how beverage items are classified in ENSIN. First, industrialized products with identifiable brand names were matched to a 2016 database containing nutritional information for packaged foods and beverages collected from 16 supermarkets representing the five largest retail chains in Bogotá [16]. Added sugar values from nutrition facts panels were linked to the corresponding branded beverages reported in ENSIN. In our supermarket and retail chain database, nutrition facts panels included added sugar values for many products. When added sugars were not explicitly reported on the label, added sugars were estimated using methods consistent with the Pan American Health Organization (PAHO) Nutrient Profile Model. Specifically, for packaged beverages that do not contain naturally occurring sugars, added sugars were assumed to be equal to total sugars. Second, homemade beverages were classified in ENSIN as disaggregated items (desglosadas) and were assigned added sugar content using ENSIN’s standardized recipe approach. These items represent beverages typically prepared at home (e.g., coffee or tea with sugar, homemade fruit juices, “aguapanela”). When a beverage was recorded as a disaggregated preparation (“desglosada”), its constituent ingredients (e.g., water, fruit, table sugar), and when available, their quantities were captured and converted to grams or milliliters using ENSIN’s standardized weights-and-measures system. To differentiate concentration and dilution across homemade preparations, ENSIN also records preparation characteristics including: (1) liquid base and proportion (water, milk, or water–milk combinations); (2) sweetener type (e.g., panela, sugar, honey) and perceived sweetness level (unsweetened, slightly sweetened, sweetened, very sweet); (3) viscosity (clear, slightly thick, thick); and for fruit beverages, (4) fruit type classified by acidity. When ingredient quantities were not available, the beverage was treated as a standardized preparation and linked to a standard recipe in the Colombian Food Composition Table (Tabla de Composición de Alimentos, TCA: https://www.icbf.gov.co/system/files/tcac_web.pdf, accessed on 17 February 2026) using these recorded characteristics to select the most appropriate standardized preparation, which helps distinguish typical dilution and sweetening patterns. For standardized preparations not available in the TCA, ENSIN reports that additional recipes were standardized by nutritionists using laboratory-based procedures informed by ingredient information elicited during the recall and the reported portion size (ENSIN 2015 [1] methodological documentation: https://www.icbf.gov.co/system/files/tcac_web.pdf (accessed on 17 February 2026). Third, beverages reported without brand information and not recorded as disaggregated preparations (“desglosadas”) were assigned nutrient values based on the standard food composition tables used by ENSIN for generic items reported without brand information (e.g., generic fruit juices such as “jugo de guayaba,” reported without brand). These reflect typical nutrient profiles for commonly consumed beverages when brand- or recipe-level detail is unavailable. This combined approach ensured that both industrialized and non-industrialized beverages were assigned added sugar content consistent with their composition, rather than assuming all carbohydrates were added sugars.

The primary basis for this classification was added sugar content, according to the regulatory thresholds defined by the tax reform by 2025. Beverages were categorized as untaxed if they had no added sugars or if they were taxed at a zero “0%” tax due to containing less than 5 g/100 mL. Beverages were categorized as taxed if they met or exceeded the threshold for the 2025 tax rate on added sugar (contained ≥5 g/100 mL). Beverages containing ≥5 and <9 g/100 mL of added sugar were taxed at 12.2%. Beverages containing equal to or more than 9 g/100 mL of added sugar were taxed at 20% [12].

Within each tax category (taxed or untaxed), beverages were further grouped based on their product type and primary ingredients. The taxed beverages were grouped into 9 categories as follows: flavored water, coffee and tea, dairy products and milk substitutes, fruit and vegetable juices, sodas and carbonated drinks, energy drinks, nutritional supplements and meal replacements, dessert beverages, and plant and grain-based beverages (Supplementary Table S1).

### 2.3. Sociodemographic Characteristics

We examined beverage intake across several sociodemographic variables, which were used in the analysis to assess differences in taxed and untaxed beverage consumption. These sociodemographic variables included sex (male, female), age (categorical, range 18–64), ethnicity (categorized as Black, Indigenous, or without ethnic recognition), population size (Four main cities of Barranquilla, Cali, Medellín, and Bogotá; Urban settlements of 100,001 to 1,000,000 inhabitants; Urban settlements of 100,000 or less inhabitants; Rest), region (Atlántico, Oriental, Orinoquia y Amazonia, Bogotá, Central, Pacifica), and income quartile. The sociodemographic data were collected through the ENSIN survey alongside the dietary data. Analyses used a complete case approach for sociodemographic covariates. 43 participants with missing ethnicity were excluded, resulting in a final analytic sample of 11,877 (Table 1).

### 2.4. Statistical Analysis

All analyses were performed using Stata 17 (StataCorp.: College Station, TX, USA, 2021; version 17) [17]. Figures were prepared in Microsoft Excel for Mac (Microsoft Corporation, Redmond, WA, USA; version 16.104.1). We estimated beverage intake from 24 h recalls using a 2-part model to account for beverage groups that have a high percentage of non-consumers, meaning study participants who did not report a particular beverage category consumed [18]. We used a probit model for the first part (likelihood of consumption). For the second part (conditional on consumption), we used a generalized linear model with a log-link and gamma distribution, which is appropriate for data that are continuous, positive, and right-skewed, typical of dietary intake data [19]. Mean volume (mL) and kilocalorie (kcal) intakes from taxed or untaxed SSBs and non-SSBs were calculated using marginal analysis, and outcomes are reported with 95% confidence intervals [20]. Estimates and confidence intervals accounted for the complex survey design, with sampling weights used for national representativeness and a linearization approach applied for variance estimation. Analyses were specified using primary sampling units, strata, and person-level weights.

Volume and kilocalorie intakes were reported per capita and per consumer, with consumers defined as any individual who reported consuming more than 0 mL of a given beverage. As a sensitivity analysis, we redefined consumers using a minimum intake threshold of ≥50 mL/day to assess whether trivial intakes influenced consumer classification. We found consumer prevalence was essentially unchanged: prevalence was 84.3% for taxed beverages and 87.5% for untaxed beverages using the primary >0 mL definition, compared with 83.8% and 86.6%, respectively, under the ≥50 mL/day threshold (differences < 1 percentage point). Confidence intervals overlapped, indicating that consumer status was not driven by very small positive intakes. To remove extreme outliers, we excluded any reported beverage intakes that were higher than six standard deviations (SD) above the mean for each beverage category. This resulted in the exclusion of 273 observations from the 131,139 total beverage observations (0.2%). Category-specific exclusions were all <1%, suggesting minimal differential trimming across beverage types. We conducted a sensitivity analysis using winsorization (capping values at the same category-specific six SD cutpoints rather than excluding observations). Results were similar for taxed and untaxed beverage volume and kilocalorie outcomes (overlapping 95% confidence intervals and no change in substantive conclusions). We kept the exclusion of higher than six SD as our primary approach for calculating beverage intakes to avoid upward bias from extreme values.

Models were adjusted for the sociodemographic variables described above. Marginal means (average predicted margins) with 95% confidence intervals were estimated for intakes of taxed and untaxed beverages, intakes of taxed and untaxed beverage subcategories, and intakes of taxed and untaxed beverages for each level of sociodemographic variables. We used post-estimation pairwise comparisons of marginal means to test differences across sociodemographic strata. For multi-level sociodemographic variables (urbanicity, income quartile, ethnicity, and region), we applied Bonferroni adjustment within each variable’s family of pairwise comparisons.

## 3. Results

### 3.1. Sample Characteristics

There was a total of 11,877 individuals in the study sample. The majority of individuals were women, representing a weighted proportion of 55.9%. Regarding ethnicity, most participants did not report any, while 7.2% of participants self-identified as Black, and 2.6% as indigenous. Participants aged 25–34 constituted the largest proportion of the sample at 25.9%, and the 55–64 age group was the smallest proportion at 11.9%. The Central region accounted for the highest weighted proportion at 23.8% of participants, and Orinoquia and Amazonia accounted for the smallest weighted proportion at 2.0%. The largest weighted group of participants (33.8%) resided in the four main cities (Barranquilla, Cali, Medellín, and Bogotá), and participants from cities with <100,000 inhabitants comprised the smallest weighted proportion at 16.1% (Table 1).

### 3.2. Overall Intake of Taxed and Untaxed Beverages

**Figure 1:**
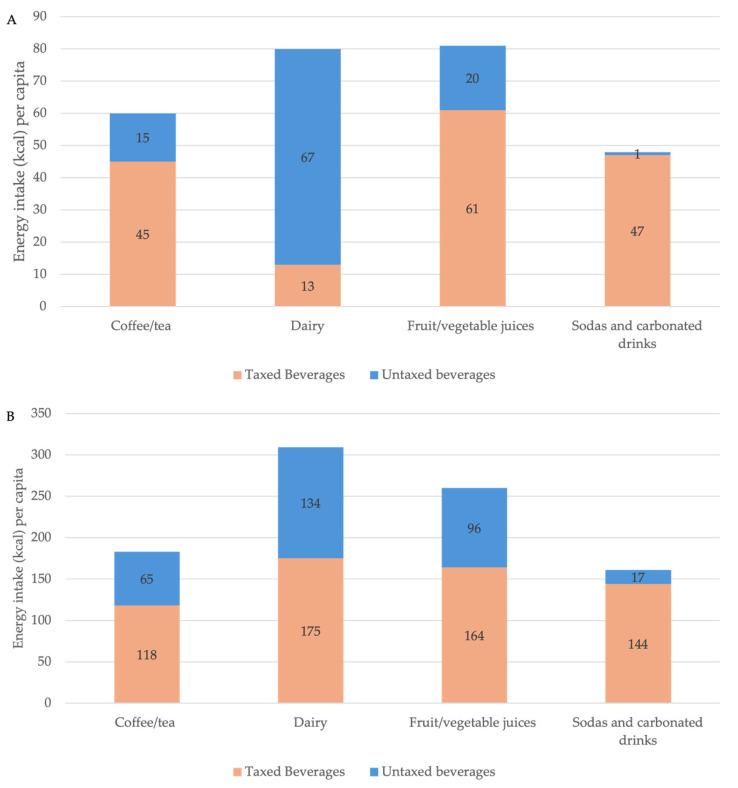
Energy intake (kcal) from taxed and untaxed beverages among beverage categories consumed by >5% of the Colombian adult population (ENSIN 2015) [1]. (**A**) Per capita intake. (**B**) Per consumer intake.

The distribution of energy intake from taxed and untaxed beverages across major beverage categories is shown in Figure 1. Beverage categories with fewer than 5% consumers were excluded for clarity and readability but are presented in Supplementary Table S2 (e.g., energy drinks, nutritional supplements and meal replacements, dessert beverages, and plant/grain-based beverages). Overall, there was high consumption of taxed beverages. Specifically, 84% of the population consumed taxed SSBs, with a per capita taxed beverage energy intake of 209 kcal/person/day (95% CI: 203–216) and a volume of 481 mL/person/day (95% CI: 469–497) and a per consumer intake of 248 kcal (95% CI: 242–254) and 570 mL (95% CI: 557–587). To add context to these mean intakes, we estimated the proportion consuming at least one and two servings of taxed beverages per day (≥250 mL and ≥500 mL). Overall, 67.1% (95% CI: 62.9–71.1) consumed ≥250 mL/day and 40.0% (95% CI: 35.9–44.2) consumed ≥500 mL/day of taxed beverages. Consumption of high-sugar taxed SSBs specifically was also high, with 63% of the population consuming them. Intake of high-sugar taxed SSBs was 134 kcal (95% CI: 130–139) and 292 mL (95% CI: 282–303) per capita and 212 kcal (95% CI: 208–217) and 461 mL (95% CI: 451–472) per consumer. In contrast, 87% of the population consumed any untaxed beverages, with a mean intake of 109 kcal (95% CI: 106–112) and 837 mL (95% CI: 793–882) per capita and 125 kcal (95% CI: 121–128) and 958 mL (95% CI: 912–1005) per consumer (Table 2).

### 3.3. Energy Intake from Taxed and Untaxed Beverage Subcategories

Fruit and vegetable juices contributed the highest energy intake among taxed beverages, with 61 kcal (95% CI: 55–66) per capita and 164 kcal (95% CI: 157–171) per consumer (Figure 1A,B). This was followed by sodas and carbonated drinks, with 47 kcal (95% CI: 44–50) per capita and 144 kcal (95% CI: 139–149) per consumer (Figure 1A,B). Coffee and tea accounted for 45 kcal (95% CI: 42–47) per capita and 118 kcal (95% CI: 115–121) per consumer (Figure 1A,B; Table 2).

Dairy products and milk substitutes contributed the highest per capita energy intake (67 kcal, 95% CI: 64–69) among untaxed beverages. Untaxed fruit and vegetable juices accounted for 20 kcal (95% CI: 18–23) per capita and 96 kcal (95% CI: 93–100) per consumer, while coffee and tea contributed 15 kcal (95% CI: 14–16) per capita and 65 kcal (95% CI: 63–68) per consumer (Figure 1A,B; Table 2).

### 3.4. Volume Intake from Taxed and Untaxed Beverage Subcategories

**Figure 2:**
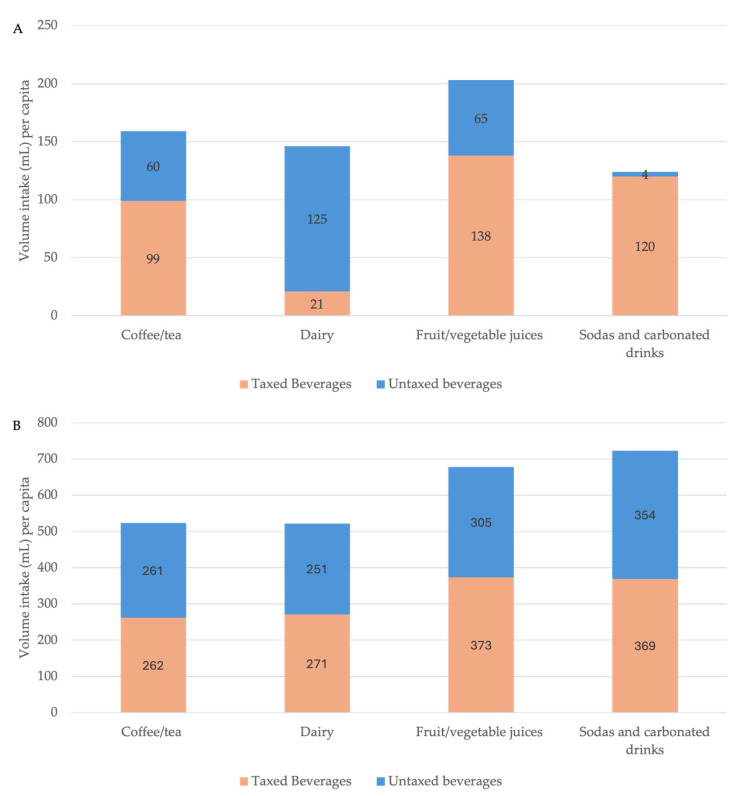
Volume intake (mL) from taxed and untaxed beverages among beverage categories consumed by >5% of the Colombian adult population (ENSIN 2015) [1]. (**A**) Per capita intake. (**B**) Per consumer intake.

Fruit and vegetable juices contributed the largest per capita volume among taxed beverages, at 138 (95% CI: 127–148) mL per capita and 373 (95% CI: 360–386) mL per consumer (Table 2). Sodas and carbonated drinks contributed 120 (95% CI: 113–128) mL per capita and 369 (95% CI: 357–382) mL per consumer, followed by coffee and tea at 99 (95% CI: 93–106) mL per capita and 262 (95% CI: 254–271) mL per consumer (Figure 2A,B).

Among untaxed beverages, dairy-based beverages contributed 125 (95% CI: 119–131) mL per capita and 251 (95% CI: 244–258) mL per consumer, while untaxed fruit and vegetable juices contributed 65 (95% CI: 58–71) mL per capita and 305 (95% CI: 294–316) mL per consumer. Untaxed coffee and tea accounted for 60 (95% CI: 56–64) mL per capita and 261 (95% CI: 247–275) mL per consumer (Figure 2A,B). Plain water accounted for by far the largest beverage volume overall, at 565 (95% CI: 521–610) mL per capita and 1052 (95% CI: 976–1128) mL per consumer (Supplementary Table S2).

### 3.5. Taxed Beverage Intake by Sociodemographic Characteristics

Males had a higher intake of taxed beverages, at 255 kcal (95% CI: 247–264) per capita (*p* < 0.001) and 291 kcal (95% CI: 282–299) per consumer, compared to females, at 174 kcal (95% CI: 165–182) per capita and 213 kcal (95% CI: 206–220). The age group with the highest intake of taxed beverages was 18–24 year-olds, at 239 kcal (95% CI: 227–251) per capita, and the lowest taxed beverage consumption was among 55–64 year-olds, at 177 kcal (95% CI: 161–194) per capita (Table 3). The lowest taxed beverage intake was observed in the first income quartile, at 193 kcal (95% CI: 183–204) per capita. Intake was significantly higher in the second quartile (220 kcal; 95% CI: 203–237; *p* = 0.001) and the third quartile (223 kcal; 95% CI: 212–233; *p* = 0.015) compared with the first quartile. The Central region had the highest taxed beverage intake, at 244 kcal (95% CI: 230–259) per capita, and Bogotá had the lowest intake, at 165 kcal (95% CI: 147–184) per capita (Table 3). Pairwise comparisons indicated that Bogotá consumed significantly less than Atlántico (189 kcal; 95% CI: 179–199; *p* = 0.036), the Central region consumed significantly more than Atlántico (*p* = 0.001), Oriental (213 kcal; 95% CI: 193–233; *p* = 0.021), and Bogotá (*p* < 0.001), and the Pacífica region (227 kcal; 95% CI: 201–253) consumed significantly more than Bogotá (*p* = 0.002). No other regional differences were statistically significant after Bonferroni adjustment (Table 3).

### 3.6. Untaxed Beverage Intake by Sociodemographic Characteristics

Males had a similar but slightly lower intake of untaxed beverages, averaging 102 kcal (95% CI: 97–108) per capita, compared to females who consumed 114 kcal (95% CI: 109–119) per capita. The age group with the highest intake of untaxed beverages was the oldest group aged 55–64, with an average intake of 106 kcal (95% CI: 92–119) per capita. The fourth income quartile had the highest intake of untaxed beverages, at 141 kcal (95% CI: 123–158) per capita, and the lowest quartile had the lowest intake at 87 kcal (95% CI: 80–95) per capita. Areas outside urban centers had the highest intake of untaxed beverages, at 115 kcal (95% CI: 101–130) per capita. The lowest intake of untaxed beverages was from the Pacifica region, at 90 kcal (95% CI: 82–99) per capita. Bogota had the highest intake of untaxed beverages, with an average intake of 142 kcal (95% CI: 131–153) per capita (Table 3).

## 4. Discussion

### 4.1. Main Findings on Taxed Beverages

This study presents an analysis of taxed and untaxed beverage intake in Colombia prior to the implementation of the SSB tax, using a survey designed to produce nationally and regionally representative estimates. A large proportion of the population consumed taxed beverages, with an average intake of 209 kcal (95% CI: 203–216) and 481 mL (95% CI: 464–497) per capita, and 248 kcal (95% CI: 242–254) and 570 mL (95% CI: 555–586) per consumer. Overall, 84% of Colombians consumed any taxed beverage, and 63% consumed beverages from the highest sugar-tax tier (≥9 g sugar/100 mL). High-sugar beverages accounted for most taxed beverage intake, indicating that Colombia’s new SSB tax is likely to affect a large share of the population, particularly those with higher sugary-drink consumption. Intake was highest among men, younger adults, and those in the middle-income quartiles, and lowest among women, older adults, and residents of Bogotá. These findings point to high baseline exposure to sugary drinks prior to the tax and identify the groups most likely to experience its greatest impact. Future evaluations will require longitudinal data linked to updated food composition tables to assess how energy and volume from taxed and untaxed beverages change after implementation.

### 4.2. Comparison of Taxed Beverage Intake to Other Latin American Countries

Our findings are consistent with previous studies in Colombia and other Latin American countries that observed high SSB consumption among adults. This study presents beverage intakes according to energy, volume, and taxation status, each of which can be compared to other Latin American countries where data are available. In terms of energy, Colombia’s overall intake of calories from beverages (323 kcal/capita/day) among adults aged 18 and older was similar but slightly lower than the pre-SSB tax beverage intake in Mexico of 382 kcal/capita/day among adults aged ≥20 y [21]. In terms of volume, Colombia’s per capita taxed beverage intake of 481 mL/day was substantially larger than findings from urban populations in Peru, which reported a per capita volume intake of approximately 93 mL/day [22]. The prevalence of taxed beverage consumption in Colombia was similar to that observed in urban Peru, with 85% of individuals in Colombia reporting consumption of beverages with ≥5 g of sugar/100 mL, and 92% of households in Peru purchasing beverages with ≥6 g of sugar/100 mL [22]. These cross-country comparisons demonstrate that although Colombia’s baseline taxed beverage intake was not the highest in Latin America, it was near pre-tax levels of the high SSB consuming country of Mexico and substantially higher than other countries with SSB tax policies like Peru. These high pre-tax levels, combined with the greater SSB tax rate implemented in Colombia relative to Latin America, suggest that the SSB tax impact is likely to be strong relative to other Latin American SSB taxes. Future evaluations should also consider the potential for substitution effects, where consumers might switch to other unhealthy options that are untaxed, which could undermine the health benefits of the policy.

### 4.3. Comparison of Beverage Subcategories to Other Latin American Countries

The types of beverages consumed also reflect patterns observed in other Latin American countries. In Colombia, the highest energy intake among taxed beverages came from fruit and vegetable juices, followed by sodas and carbonated drinks, while untaxed beverages were primarily dairy-based drinks and coffee or tea. These findings align with prior studies from Brazil, where a national dietary survey found that caloric coffee beverages were the most commonly consumed [23]. The three most consumed taxed beverage subcategories in Colombia were similar to those in Mexico, where caloric sodas, caloric coffee/tea, and agua fresca (a sweetened fruit drink) were the largest contributors to energy intake from beverages prior to SSB tax implementation [21]. Intakes of caloric sodas were similar but slightly lower than in Mexico, at 124 mL/capita/day and 50 kcal/capita/day in Colombia and 201 mL/capita/day and 87 kcal/capita/day in Mexico [21]. The prevalence of soda consumption was also similar, with 34% of Colombian adults consuming taxed caloric soda compared to 42% of Mexican adults consuming caloric sodas [21]. Caloric coffee/tea was also consumed at nearly identical rates between the two countries, with 40% of Colombians consuming taxed coffee/tea compared to 38% of Mexicans consuming calorie coffee/tea [21].

These similarities suggest that baseline beverage intake in Colombia is similar to that of Mexico prior to its SSB tax, providing useful context for anticipating potential effects of the Colombian tax. In Mexico, reductions in taxed beverage purchases were modest in the first year but grew larger in the second year [24]. Given Colombia’s higher tax rate of up to 20%, the policy may produce even greater reductions in high-sugar beverage intake if consumer responses follow comparable patterns.

### 4.4. Beverage Intake by Sociodemographic Characteristics

Our findings are consistent with previous studies in Colombia and other Latin American countries that have observed high SSB consumption among adults. Using a survey designed to produce nationally and regionally representative estimates, we identified clear differences in beverage intake across sociodemographic groups. Taxed beverage intake was highest among men, younger adults, and those in the middle-income quartiles, and lowest among women, older adults, and residents of Bogotá. These patterns are consistent with previous research in Peru and Brazil, where SSB consumption was also highest among males and younger adults and lowest among those with lower income or education levels [22,23]. The results from this study differ slightly from those in Mexico for income. In Mexico, pre-tax SSB purchases were highest among low-income households, and they decreased the most after the SSB tax was implemented [25]. In Colombia, a lower baseline intake among low-income groups and higher intake among middle-income groups suggest that the tax’s immediate health benefits may be more evenly distributed or concentrated among middle-income populations, rather than strongly progressive as seen in Mexico. Monitoring consumption changes across income groups will be important to assess whether the policy reduces or reinforces socioeconomic disparities in SSB consumption.

We observed variation in taxed beverage intake across different sociodemographic groups, with men, young adults (18–24 years), and the two middle-income quartiles consuming the most SSBs. Mean energy intake from taxed beverages was slightly lower in our study (186 kcal/day for women and 272 kcal/day for men) compared to another study of urban populations in Colombia (287 kcal/day for women and 334 kcal/day for men), but both studies found similar differences in intake by sex [14]. Taken together, these two findings suggest a consistent difference in taxed beverage intake by sex in Colombia. Our findings by sociodemographic characteristics are also consistent with data from other Latin American countries [26], including Peru and Brazil, where SSB consumption is highest among males and younger adults, and lowest among groups with lower income or education levels [23,27]. These patterns suggest that Colombia’s SSB tax may have the greatest impact among these highest consuming groups. Future evaluations should examine how intake among these groups changes relative to other groups and consider whether complementary policies are needed to further reduce taxed beverage intake.

The analysis by population density revealed that Bogotá had the lowest per capita intake of taxed beverages, while it had the highest consumption of untaxed beverages. This finding may be explained by the education level and other welfare indicators of this city, which are the highest in Colombia [28]. Additionally, public health advocacy and debate surrounding the health risks of SSB consumption have occurred in Bogotá as early as 2009. A 2011 study identified how the SSB industry sponsored physical activity programs in Bogotá to increase political influence and prevent SSB regulations [29]. These early and visible discussions around the harms of sugary drinks may have raised public awareness regarding the health consequences of SSB consumption in Bogotá, helping to explain the lower taxed beverage intake we observed. Therefore, it is possible that the SSB tax impact in Bogotá could be modified by baseline public awareness of health risks [30]. Future studies should assess whether individual factors, such as knowledge of SSB health risks, modify behavioral responses to the tax [30]. Evaluations should also assess whether areas such as Bogotá experience greater reductions due to heightened awareness or lesser reductions due to lower baseline consumption.

We also identified regional variation, with the highest taxed beverage intake in the Central and Pacífica regions. Differences based on population size were small, suggesting that high SSB intake is widespread across Colombia. These findings highlight socioeconomic and regional gradients in beverage intake that are relevant for evaluating the SSB tax, and future evaluations should track how these patterns change over time.

### 4.5. Implications for Policy

The findings of this study, showing very high consumption of SSBs in Colombia, demonstrate the importance of evaluating the impact of the newly implemented SSB tax. Similar policies in other countries have led to meaningful reductions in taxed beverage intake. For example, Mexico’s SSB tax, which is set at 10% and approximately half of the high-sugar SSB tax category in Colombia, was associated with a 6% reduction in taxed beverage purchases in the first year, with larger decreases among lower-income households [31]. In Chile, a tiered tax that increased from 13% to 18% for high-sugar beverages led to a 3.4% reduction in the volume of high-sugar SSB purchases and a 4.0% decrease in the associated caloric intake [32]. Reductions were greater among higher socioeconomic status households, which saw a 6.4% decrease in volume and a 6.5% decrease in calories from high-sugar SSBs [32]. Colombia’s SSB tax is one of the highest in the region, with a top rate of 20% on high-sugar beverages that aligns with international recommendations [33,34], which could lead to even larger reductions. Despite this strength, Colombia’s SSB tax remains below recent World Health Organization (WHO) recommendations to increase the real prices of tobacco, alcohol, and sugary drinks by at least 50% by 2035 [35]. The tax was phased in from 2023 to 2025, and the extent to which this approach may dampen the immediate effects of the tax will need to be evaluated.

An implication of Colombia’s SSB tax being based on sugar concentration is that it may reduce sugar intake through both consumer responses via lower consumption and industry responses via product reformulation. However, a possible unintended consequence of taxes based on sugar content is that they can incentivize substitution toward reformulated products that use non-nutritive sweeteners. Indeed, there is evidence of increased availability and purchases of beverages containing non-nutritive sweeteners after policies that target sugar concentration in the United Kingdom and Chile [36,37]. WHO guidance emphasizes the importance of anticipating and monitoring substitution and other unintended consequences when designing and evaluating SSB taxes, including shifts between taxed and untaxed products and changes in sweetener use [38].

This baseline analysis of taxed beverage intake in Colombia prior to SSB tax implementation provides a reference point for future evaluations of the tax impact. Evaluations should examine changes in taxed beverage intake using the sugar thresholds in effect during each policy year, examine substitutions of untaxed beverages for taxed beverages, and test whether responses to the tax differ by the higher intake groups identified in this analysis. Finally, future evaluations must account for industry responses to the tax, including reformulation effects, using food composition tables that are updated over time [39].

### 4.6. Strengths and Limitations

This study has several strengths, including the use of a survey designed to produce nationally and regionally representative estimates of dietary intake, allowing us to examine beverage consumption patterns across the Colombian population. These results describe beverage consumption across a wide range of demographic characteristics in the Colombian population, and they are timely, as they provide a baseline description of beverage consumption patterns in Colombia that preceded the SSB tax in 2023. By examining intake across various sociodemographic factors, the study can be used to identify and highlight key disparities in taxed beverage consumption in the Colombian population, which could inform targeted public health interventions.

This study also has limitations. The use of a single year of intake means that we can only describe beverage consumption patterns as they existed in 2015, and future studies will require more data to characterize changing trends in beverage consumption in Colombia. Although ENSIN captures both industrialized branded beverages and home-prepared beverages (recorded as disaggregated “desglosadas” items described in the Methods), some unbranded or out-of-home beverage reports lack sufficient detail to consistently classify preparation source (industrialized vs. prepared at home). The dietary intake data used in this study were collected via 24 h recalls, which are potentially subject to social desirability bias and recall bias. Social desirability bias could cause people to underreport sugary beverage intake. However, this survey was conducted before the tax, and people would not have a significant policy signal that would cause them to underreport a taxed beverage. Using 24 h recalls from a single day introduces some risk of misclassifying episodic consumers as non-consumers, which could underestimate consumer prevalence for specific beverage categories. If episodic consumption differs across beverage categories, this may affect comparisons between taxed and untaxed groups. However, 24 h recalls are considered suitable for estimating population means intake in large surveys because within-person random errors typically average out at the group level, leading to potentially reduced precision rather than systematic bias in the mean [15]. Future evaluations should utilize repeated dietary intake or purchase data to quantify changes in both the prevalence of consumption and consumption levels among consumers.

## 5. Conclusions

This study provides baseline estimates of beverage consumption in Colombia using 2015 intake data collected prior to implementation of the country’s tiered, sugar concentration-based SSB tax, offering a practical estimate of the tax’s intended coverage. Per capita energy intake was higher from taxed than untaxed beverages, while per capita volume was higher for untaxed beverages, driven largely by plain water consumption. Taxed beverage intake was highest among men, young adults, and the two middle-income quartiles. These results show which beverage types and population groups account for the most taxed beverage intake, which can help policymakers focus implementation and monitoring efforts on those with the highest intake. Even without post-implementation data, identifying population groups with higher taxed beverage intake can inform policy and practice because it can help prioritize complementary policies and targeted communication efforts among groups with the highest taxed beverage intake. Future evaluations are needed to track changes in overall taxed SSB consumption and disparities in consumption across time.

## Figures and Tables

**Table 1:** Sociodemographic characteristics of the National Nutrition Survey for Colombia, 2015 (total *n* = 11,877).

Variable	*n*	Weighted %
**Sex**		
Male	4572	43.7%
Female	7305	56.2%
**Age**		
18–24	2700	20.7%
25–34	3136	25.7%
35–44	2309	21.3%
45–54	2212	20.1%
55–64	1520	12.1%
**Ethnicity**		
Majority	10,146	90.0%
Black	988	7.3%
Indigenous	743	2.7%
**Population size**		
Four main cities (Barranquilla, Cali, Medellín, and Bogotá)	1680	33.3%
Urban settlements of 100,001 to 1,000,000 inhabs.	3284	30.1%
Urban settlements of 100,000 or less inhabs.	4136	16.2%
Other	2777	20.4%
**Region**		
Atlántico	2681	21.5%
Oriental	2131	16.7%
Bogota	830	17.3%
Orinoquia & Amazonia	1804	2.1%
Central	2825	23.7%
Pacifica	1606	18.6%

The estimated sampling frame of the survey was 26,821,570. Bolded text indicates category headings within the table.

**Table 2:** Weighted adjusted mean intakes for beverage categories and subcategories.

Beverage Categories	Per Capita		Per Consumer		% Consumers
	kcal (95% CI)	Volume (mL) (95% CI)	kcal (95% CI)	Volume (mL) (95% CI)	
**High-Sugar Taxed Beverages**	134 (130–139)	292 (282–303)	212 (208–217)	461 (451–472)	63
**Taxed Beverages**	209 (203–216)	481 (464–497)	248 (242–254)	570 (555–586)	84
Flavored Waters	34 (30–38)	84 (75–93)	148 (141–156)	367 (349–385)	23
Coffee/tea	45 (42–47)	99 (93–106)	118 (115–121)	262 (254–271)	38
Dairy	13 (11–15)	21 (18–23)	175 (167–183)	271 (263–278)	8
Fruit/vegetable juices	61 (55–66)	138 (127–148)	164 (157–171)	373 (360–386)	37
Sodas and carbonated drinks	47 (44–50)	120 (113–128)	144 (139–149)	369 (357–382)	33
**Untaxed beverages**	109 (106–112)	837 (793–882)	125 (121–128)	958 (912–1005)	87
Plain water	0	565 (520–609)	0	1052 (976–1127)	54
Coffee/tea	15 (14–16)	60 (56–64)	65 (63–68)	261 (247–275)	23
Dairy	67 (64–69)	125 (119–131)	134 (130–137)	251 (244–258)	50
Fruit/vegetable juices	20 (18–23)	65 (58–71)	96 (93–100)	305 (294–316)	21

Beverage categories with fewer than 5% consumers were excluded from this table for clarity and readability. These categories include Energy Drinks, Nutritional Supplements and Meal Replacements, Dessert Beverages, Plant/Grain-Based Beverages, untaxed flavored waters, and untaxed sodas and carbonated drinks. Bolded text indicates category headings within the table.

**Table 3:** Weighted adjusted mean intakes for beverage categories by sociodemographic groups per capita.

Sociodemographic Variable	Taxed		Untaxed	
	kcal (95% CI)	Volume (mL) (95% CI)	kcal (95% CI)	Volume (mL) (95% CI)
**Sex**				
Male	255 (247–264)	585 (565–604)	102 (97–108)	872 (815–928)
Female	174 (165–182)	400 (378–421)	114 (109–119)	811 (752–870)
**Age**				
18–24	239 (227–251)	536 (509–564)	116 (107–124)	843 (770–916)
25–34	222 (209–235)	506 (478–534)	108 (100–115)	849 (798–899)
35–44	198 (182–214)	458 (421–495)	111 (95–126)	858 (768–948)
45–54	194 (183–205)	454 (435–473)	105 (95–115)	809 (753–866)
55–64	177 (161–194)	414 (374–454)	106 (92–119)	815 (751–879)
**Income Quartile**				
1st quartile	193 (183–204)	453 (427–478)	87 (80–95)	798 (723–874)
2nd quartile	220 (203–237)	512 (467–557)	101 (92–110)	829 (750–909)
3rd quartile	223 (212–233)	502 (480–525)	111 (98–123)	819 (737–902)
4th quartile	203 (193–214)	458 (434–483)	141 (123–158)	907 (846–968)
**Population size**				
Four main cities (Bogotá, Barranquilla, Cali, Medellin)	209 (185–233)	483 (429–537)	106 (98–114)	780 (684–876)
Medium urban settlements *	220 (197–222)	479 (448–511)	113 (103–123)	888 (821–955)
Small urban settlements *	208 (189–227)	476 (437–514)	103 (94–111)	903 (820–986)
Rest	211 (193–230)	485 (439–531)	115 (101–130)	777 (688–867)
**Region**				
Atlántico	189 (179–199)	434 (411–458)	95 (89–102)	1429 (1311–1547)
Oriental	213 (193–233)	492 (441–542)	117 (105–128)	592 (502–682)
Bogota	165 (147–184)	378 (337–418)	142 (131–153)	643 (578–708)
Orinoquia & Amazonia	217 (188–246)	473 (414–532)	98 (84–111)	421 (239–603)
Central	244 (230–259)	561 (528–583)	107 (98–115)	720 (601–839)
Pacifica	227 (201–253)	523 (457–588)	90 (82–99)	755 (628–882)

* Medium and small urban settlements refer to sizes of 100,001 to 1,000,000 inhabitants and 100,000 or fewer inhabitants, respectively. Bolded text indicates category headings within the table.

## Data Availability

This study used anonymized data from the 2015 National Survey of Nutritional Status in Colombia (ENSIN 2015) [1]. This data is accessible to researchers through an online request in the Microdata of surveys and population studies provided by the Ministry of Health and Social Protection of Colombia (https://enlinea.minsalud.gov.co/Encuestas/Microdatos.aspx?E=ENSIN2015, accessed on 17 February 2026). Access to the portal may be restricted by country/IP address for some users outside Colombia. The Ministry’s “Estudios y encuestas” page provides an alternative entry point and links to ENSIN resources (https://www.minsalud.gov.co/salud/publica/epidemiologia/Paginas/Estudios-y-encuestas.aspx, accessed on 17 February 2026).

## Supplementary Materials

The following supporting information can be downloaded at https://www.mdpi.com/article/10.3390/nu18050716/s1: Table S1: Beverage categorization system used in analysis; Table S2: Weighted adjusted mean intakes for beverage categories and subcategories, all categories included.

## Abbreviations

The following abbreviations are used in this manuscript:

SSBs	Sugar-sweetened beverages
ENSIN	National Survey of the Nutritional Situation in Colombia (Encuesta Nacional de la Situación Nutricional)
