# Supplementary material for: Consumption of Sugary Beverages by Adults Prior to Sugary Drink Tax in Colombia: An Analysis of the National Nutrition Survey 2015
Source: Nutrients. 2026 Feb 24;18(5):716. doi: 10.3390/nu18050716 (PMC12986462; doi:10.3390/nu18050716)
Supplement: Supplementary file 1 [file nutrients-18-00716-s001.zip › nutrients-4051300-supplementary.pdf]

Supplementary Table S1. Beverage categorization system used in analysis

Beverage group	Description	Examples
Taxed SSB	Meet conditions for taxation based on levels of added sugars (($\geq$5g sugar/100mL)	
Flavored Waters	Includes industrialized flavored water with added sugar that crosses regulation thresholds.	Agua de panela
Coffee/tea	All coffee beverages, including any beverage that contains instant, powder, ground, roasted coffee not likely to cross sugar threshold. All tea beverages, including regular and herbal teas, ground tea, tea bags, to brew or ready-to-drink powder likely to cross sugar threshold.	Nestea de limón, Té de Lipton
Dairy, milk substitutes, and drinkable yogurts	Includes flavored, unflavored, and powdered milk, dairy drinks, drinkable yogurts, non-dairy milk likely to cross sugar threshold.	Yox de Alpina, Chocolisto en leche con azúcar
Fruit/vegetable juices	Includes fruit/vegetable juices, fruit juice concentrates, nectar likely to cross sugar threshold.	Jugo de guayaba con azúcar
Sodas and carbonated drinks	Includes regular sugar sodas and, other broader sugary drinks likely to cross sugar threshold.	Gaseosa Mountain Dew, Pony Malta
Energy drinks	Includes sports drinks, ready-to-drink energy drinks and powdered energy drinks likely to cross sugar threshold.	Red Bull
Nutritional supplements and meal replacement beverages	Includes powdered supplements and beverages intended to satisfy nutritional needs for age and stage of life likely to cross sugar threshold.	Ensure
Dessert beverages	Includes beverages such as milkshakes, frappés, and smoothies likely to cross sugar threshold.	Malteada de helado de fresa y vanilla
Plant/grain based beverages	Includes beverages made of grains, seeds, and root vegetables likely to cross sugar threshold.	Colada de avena, colada de arroz, chicha de maiz, batido de linaza, bebida a base de harina de yuca fermentada
Untaxed SSB	Do not meet conditions for regulation based on levels of added sugar	
Coffee/tea	All coffee beverages, including any beverage that contains instant, powder, ground, roasted coffee not likely to cross sugar threshold. All tea beverages, including regular and herbal teas, ground tea, tea bags, to brew or ready-to-drink powder not likely to cross sugar threshold.	Café con leche y azúcar, tinto con azúcar, aromática
Dairy, milk substitutes, and drinkable yogurts	Includes flavored, unflavored, and powdered milk, dairy drinks, drinkable yogurts, non-dairy milk not likely to cross sugar threshold.	Leche de vaca líquida deslactosada

Fruit/vegetable juices	Includes fruit/vegetable juices, fruit juice concentrates, not likely to cross sugar threshold.	Jugo de guayaba, jugo de tomate de árbol, nectar de pera
Sodas and carbonated drinks	Includes regular sugar sodas and, other broader sugary drinks not likely to cross sugar threshold.	Coca-cola Zero
Energy drinks	Includes sports drinks, ready-to-drink energy drinks and powdered energy drinks not likely to cross sugar threshold.	
Nutritional supplements and meal replacement beverages	Includes powdered supplements and beverages intended to satisfy nutritional needs for age and stage of life not likely to cross sugar threshold.	Batido de proteina Quest, Bienestarina,
Dessert beverages	Includes beverages such as milkshakes, frappés, and smoothies not likely to cross sugar threshold.	
Plant/grain based beverages	Includes beverages made of grains, seeds, and root vegetables not likely to cross sugar threshold.	Colada de arroz, chicha de maiz, batido de linaza, bebida a base de harina de yuca fermentada

Bolded text indicates category headings within the table.

Supplementary Table S2. Weighted adjusted mean intakes for beverage categories and subcategories, all categories included.

Beverage categories	Per Capita kcal (95% CI)	Per Capita volume (mL) (95% CI)	Per Consumer kcal (95% CI)	Per Consumer volume (mL) (95% CI)	% Consumers
**High-Sugar Taxed Beverages**	134 (130-139)	292 (282-303)	212 (208-217)	461 (451-472)	63
**Taxed Beverages**	209 (203-216)	481 (464-497)	248 (242-254)	570 (555-586)	84
Flavored Waters	34 (30-38)	84 (75-93)	148 (141-156)	367 (349-385)	23
Coffee/tea	45 (42-47)	99 (93-106)	118 (115-121)	262 (254-271)	38
Dairy	13 (11-15)	21 (18-23)	175 (176-183)	271 (263-278)	8
Fruit/vegetable juices	61 (55-66)	138 (127-148)	164 (157-171)	373 (360-386)	37
Sodas and carbonated drinks	47 (44-50)	120 (113-128)	144 (139-149)	369 (357-382)	33
Energy drinks	2 (2-3)	7 (5-8)	147 (134-160)	448 (411-485)	2
Nutritional supplements and meal replacements	2 (1-2)	2 (1-3)	212 (174-249)	316 (282-350)	1
Dessert beverages	2 (1-3)	3 (2-3)	234 (194-274)	318 (264-372) 8	1
Plant/grain-based beverages	4 (3-4)	6 (5-8)	151 (141-162)	254 (238-269)	2
**Untaxed beverages**	109 (106-112)	837 (793-882)	125 (121-128)	958 (912-1005)	87
Plain water	0 (0-0)	565 (521-610)	0 (0-0)	1052 (976-1128)	54
Coffee/tea	15 (14-16)	60 (56-64)	65 (63-68)	261 (247-275)	23
Dairy	67 (64-69)	125 (119-131)	134 (130-137)	251 (244-258)	50
Fruit/vegetable juices	20 (18-23)	65 (58-71)	96 (93-100)	305 (294-316)	21
Sodas and carbonated drinks	1 (0-2)	4 (3-5)	17 (12-21)	354 (327-378)	0.01
Energy drinks	0 (0-0)	0 (0-0)	0 (0-0)	0 (0-0)	0
Nutritional supplements and meal replacement beverages	1 (0-1)	2 (1-2)	80 (4-155)	262 (45-480)	1
Dessert beverages	5 (4-6)	9 (7-10)	194 (183-205)	328 (309-347) 0	3
Plant/grain-based beverages	1(1-1)	3 (2-4)	76 (62-90)	271 (212-329)	1

Bolded text indicates category headings within the table.
